# Supplementary material for: Qualitative and Quantitative Analysis of Edible Bird’s Nest Based on Peptide Markers by LC-QTOF-MS/MS
Source: Molecules. 2022 May 5;27(9):2945. doi: 10.3390/molecules27092945 (PMC9104280; doi:10.3390/molecules27092945)
Supplement: Supplementary file 1 [file molecules-27-02945-s001.zip › molecules-1648320-supplementary.pdf]

Supplementary Materials

# Qualitative and quantitative analysis of Edible Bird's Nest based on peptide markers by LC-QTOF-MS/MS

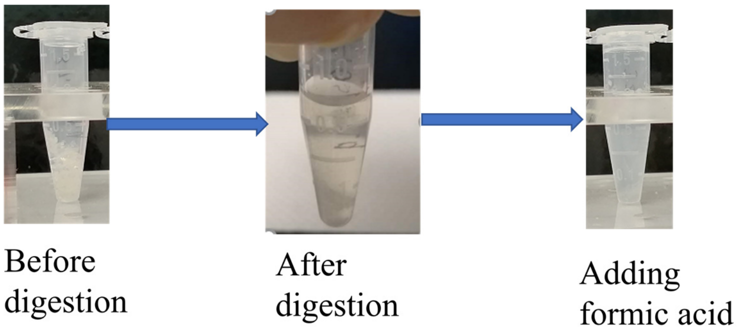


**Figure S1.** Edible bird's nest is fully soluble after adding formic acid.

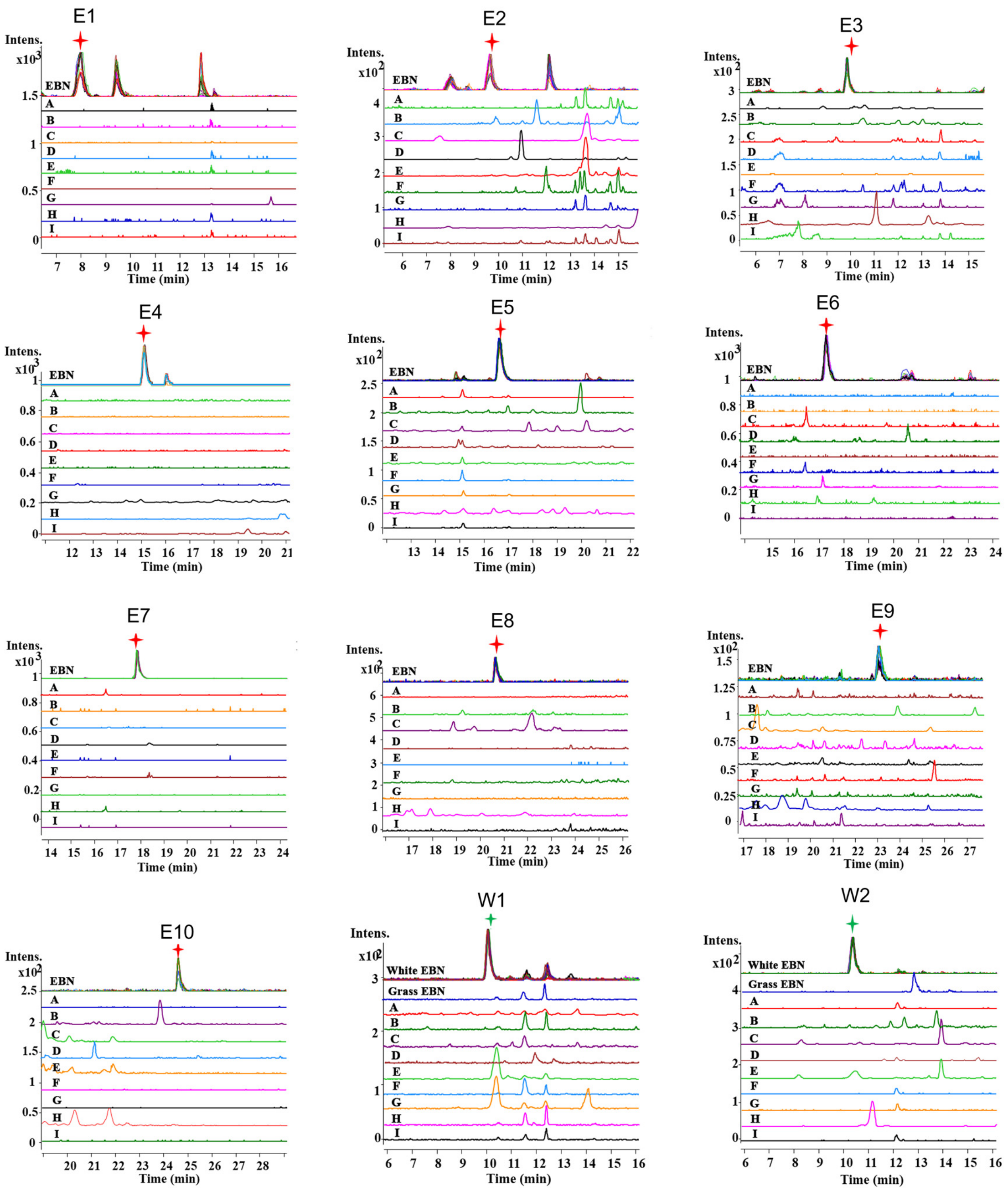
E1
Intens. x10³
EBN
A
B
C
D
E
F
G
H
I
Time (min)
E2
Intens. x10²
EBN
A
B
C
D
E
F
G
H
I
Time (min)
E3
Intens. x10²
EBN
A
B
C
D
E
F
G
H
I
Time (min)
E4
Intens. x10³
EBN
A
B
C
D
E
F
G
H
I
Time (min)
E5
Intens. x10²
EBN
A
B
C
D
E
F
G
H
I
Time (min)
E6
Intens. x10³
EBN
A
B
C
D
E
F
G
H
I
Time (min)
E7
Intens. x10³
EBN
A
B
C
D
E
F
G
H
I
Time (min)
E8
Intens. x10²
EBN
A
B
C
D
E
F
G
H
I
Time (min)
E9
Intens. x10²
EBN
A
B
C
D
E
F
G
H
I
Time (min)
E10
Intens. x10²
EBN
A
B
C
D
E
F
G
H
I
Time (min)
W1
Intens. x10²
White EBN
Grass EBN
A
B
C
D
E
F
G
H
I
Time (min)
W2
Intens. x10²
White EBN
Grass EBN
A
B
C
D
E
F
G
H
I
Time (min)

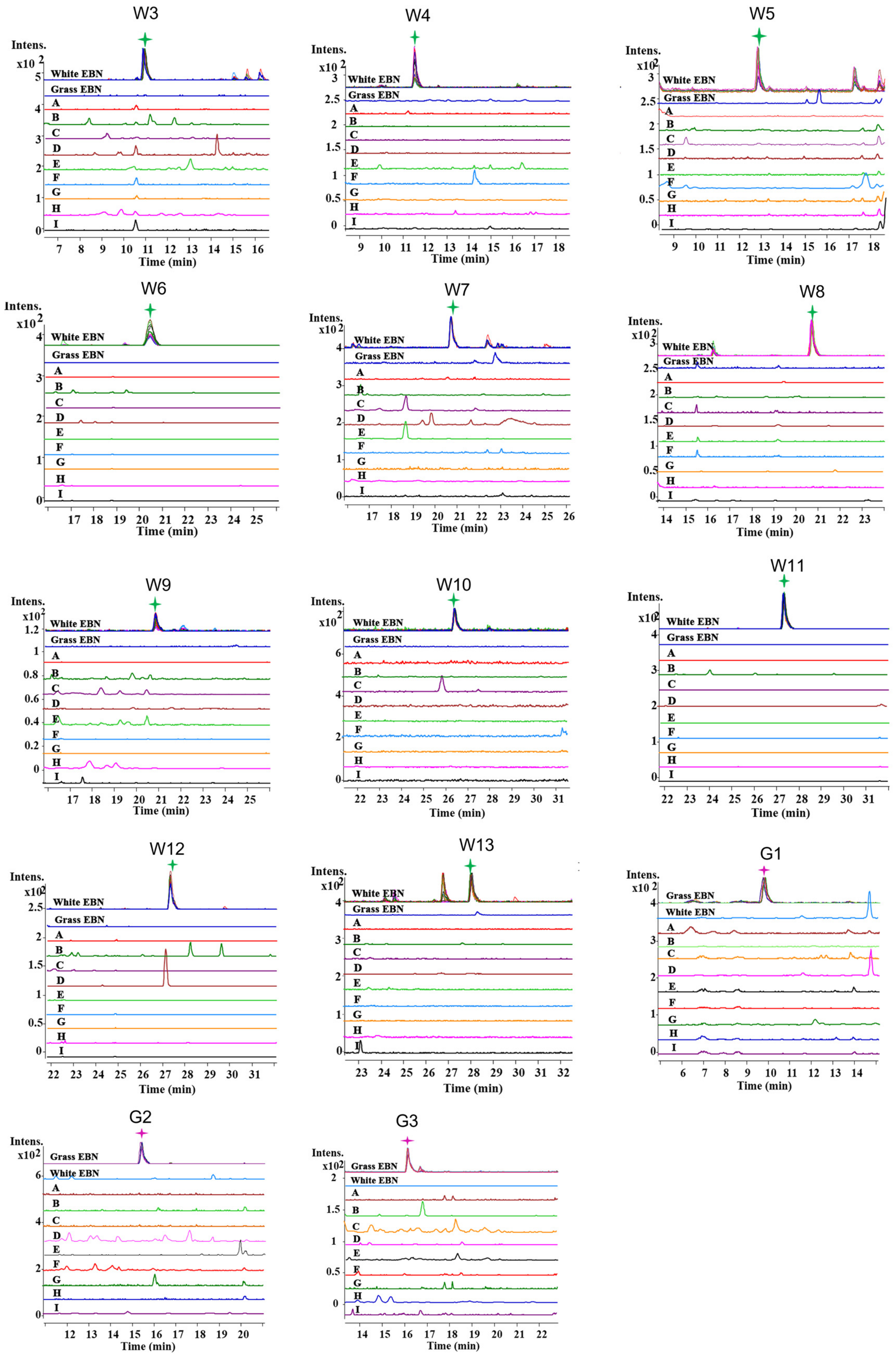


**Figure S2.** Extracted ion chromatograms (EIC) of selected peptide markers specific to EBN, white EBN and grass EBN in typical batches of digested EBN (overlapped) and related adulterants. The adulterants include agar (A), egg white (B), gelatin (C), cow milk (D), pork skin (E), rice flour (F), starch (G), swim

bladder (H) and tremella fungus (I).

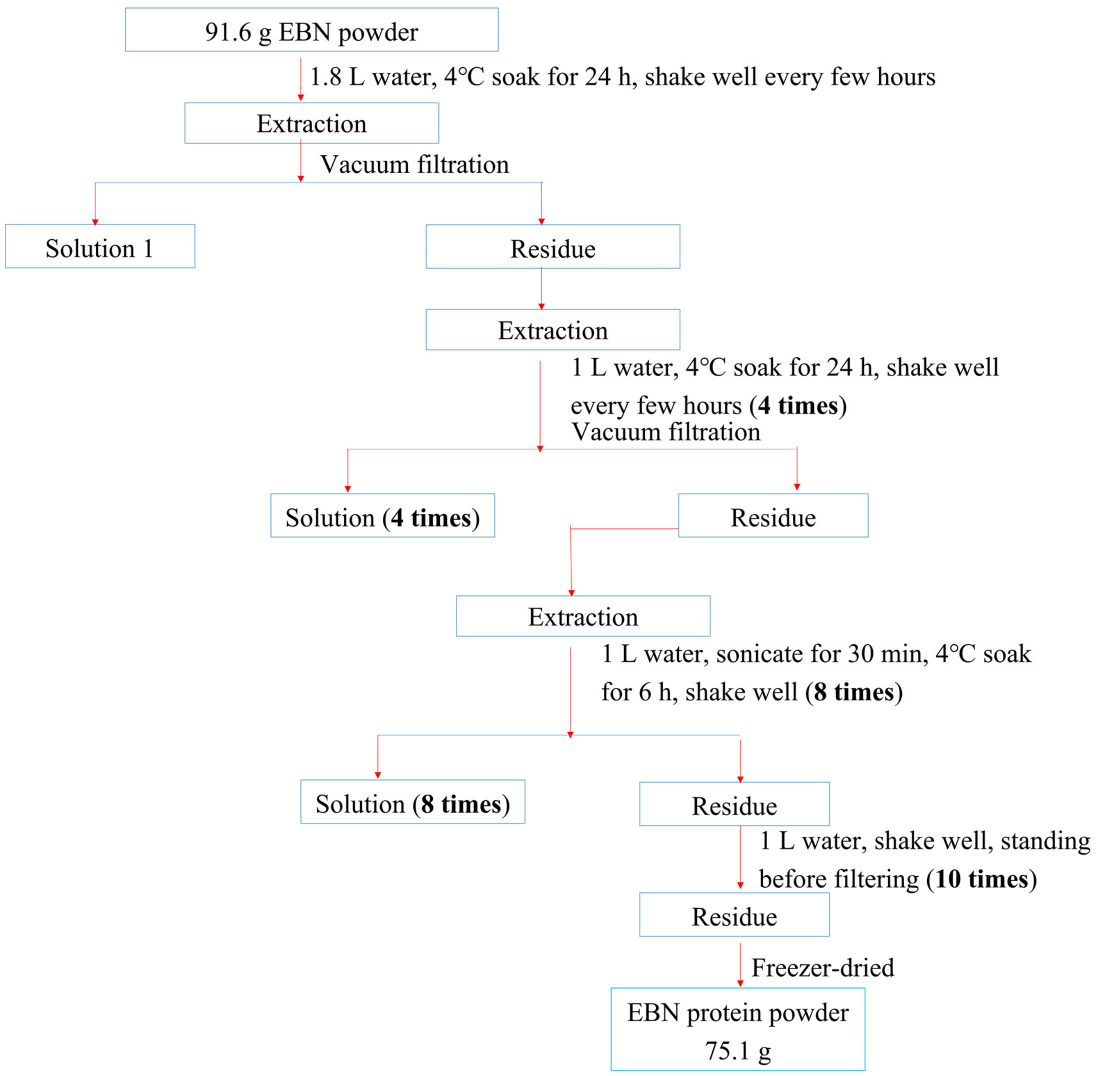


**Figure S3.** Flowchart for homemade EBN total protein.

**Table S1.** peak areas of peptide markers in the same sample.

| Marker No. | Mass to charge (m/z) | Retention time (min) | Peak area in raw material | Peak area in supernatant | Peak area in residue |
|---|---|---|---|---|---|
| W2 | 454.7018 | 10.24 | 3116011 | 1131499 | 7476481 |
| W4 | 277.6512 | 11.50 | 21259 | 107630 | 157269 |
| E5 | 404.1986 | 16.65 | 107159 | 178887 | 226653 |
| W6 | 321.7031 | 20.50 | 1824927 | 1005215 | 1400215 |
| W9 | 468.7439 | 20.78 | 2877560 | 1526356 | 2610093 |
| W11 | 391.7427 | 27.42 | 5808 | 9038 | 8829 |
